# Development of Taste Sensor with Lipid/Polymer Membranes for Detection of Umami Substances Using Surface Modification

**DOI:** 10.3390/bios14020095

**Published:** 2024-02-11

**Authors:** Wenhao Yuan, Zeyu Zhao, Shunsuke Kimura, Kiyoshi Toko

**Affiliations:** 1 Graduate School of Information Science and Electrical Engineering, Kyushu University, 744 Motooka, Nishi-ku, Fukuoka 819-0395, Japan; yuan.wenhao.461@s.kyushu-u.ac.jp (W.Y.); zeyu.zhao.720@s.kyushu-u.ac.jp (Z.Z.); 2 Research and Development Center for Five-Sense Devices, Kyushu University, 744 Motooka, Nishi-ku, Fukuoka 819-0395, Japan; 3 Institute for Advanced Study, Kyushu University, 744 Motooka, Nishi-ku, Fukuoka 819-0395, Japan

**Keywords:** taste sensor, umami measurement, lipid/polymer membrane, surface modification

## Abstract

A taste sensor employs various lipid/polymer membranes with specific physicochemical properties for taste classification and evaluation. However, phosphoric acid di(2-ethylhexyl) ester (PAEE), employed as one of the lipids for the taste sensors, exhibits insufficient selectivity for umami substances. The pH of sample solutions impacts the dissociation of lipids to influence the membrane potential, and the response to astringent substances makes accurate measurement of umami taste difficult. This study aims to develop a novel taste sensor for detecting umami substances like monosodium L-glutamate (MSG) through surface modification, i.e., a methodology previously applied to taste sensors for non-charged bitter substance measurement. Four kinds of modifiers were tested as membrane-modifying materials. By comparing the results obtained from these modifiers, the modifier structure suitable for measuring umami substances was identified. The findings revealed that the presence of carboxyl groups at para-position of the benzene ring, as well as intramolecular H-bonds between the carboxyl group and hydroxyl group, significantly affect the effectiveness of a modifier in the umami substance measurement. The taste sensor treated with this type of modifier showed excellent selectivity for umami substances.

## 1. Introduction

Taste sensing systems, a category of chemical sensors, have received much attention in recent years due to their objectivity and reproducibility, which offer important economic benefits to food industries [1,2,3,4,5] and pharmaceutical industries [6,7,8]. These systems encompass a range of sensing methodologies [9,10], such as electrochemical methods [11,12,13,14,15,16,17,18,19,20,21] (e.g., potentiometry or voltammetry), biomimetic biosensing [22,23], optical methods [24,25], mass change sensing methods [26] or enzymatic methods [27,28,29].

A potentiometry taste sensor, developed by Toko and other co-workers [30], has been commercially used in food industry [31,32,33,34], beverages [35,36,37,38], and medicine [39,40,41,42,43,44]. An example of commercialized taste sensing system utilizing potentiometric measurement is TS-5000Z (Intelligent Sensor Technology, Atsugi, Japan). This system features multichannel sensor electrodes composed of various types of lipid/polymer membranes to measure the basic five tastes (saltiness, sourness, umami, bitterness, and sweetness) and astringency [45,46,47,48]. For example, CT0 sensor is used to measure saltiness, CA0 sensor for sourness, AAE sensor for umami, C00 sensor and BT0 sensor for bitterness, GL1 sensor for sweetness, and AE1 sensor for astringency [46].

Taste sensors enabled the classification and quantification of the five basic tastes based on a method that involves designing lipid/polymer membranes according to the physiochemical properties of taste substances, such as electric charge and hydrophobicity [45,46,48]. For example, charged bitter substances with high hydrophobicity can be adsorbed onto the membrane through electrostatic and hydrophobic interactions, thereby causing changes in membrane potential. Sour substances or salty substances induce a change in membrane potential through hydrophilic ions interacting with the membrane. In other words, the amphiphilic lipid content is designed to be minimized in a bitterness sensor to increase hydrophobicity, while a sourness sensor or a saltiness sensor has a membrane with a higher proportion of amphiphilic lipid to increase hydrophilicity, promoting more electrostatic interactions with ions. Nevertheless, this approach is unsuitable for measuring non-charged substances because the system detects changes in electric potential due to change in the membrane surface charge density caused by charged taste substances.

Yoshimatsu et al. [49] have reported a novel method to measure non-charged bitter substances utilizing membrane surface modification on lipid/polymer membranes. This measurement relies on allostery, i.e., a phenomenon occurring in many enzymes and receptors including taste receptors, where binding to a ligand at one site affects binding to a different or the same type of ligand at another distant site of the enzyme or receptor molecule [50,51,52]. The sensor electrodes were immersed into the solution of 2,6-dihydroxybenzoic acid (2,6-DHBA), allowing ionized 2,6-DHBA to be adsorbed onto the lipid/polymer membrane surface through hydrophobic interactions. These fabricated electrodes were employed in the measurement of non-charged bitter substances like caffeine [49,53]. Within this modified membrane, 2,6-DHBA is ionized under a wide range of pH, forming intramolecular H-bond. The ionized 2,6-DHBA can interact with caffeine through its hydroxyl groups [54]. This interaction is achieved by breaking intramolecular H-bonds, forming intermolecular H-bonds with caffeine by taking back dissociated H^+^ from the solution. Consequently, this returned H^+^ leads to an increase in surface charge density, resulting in an increase in membrane potential. The binding of 2,6-DHBA with caffeine has been confirmed by ^1^H-NMR measurement [54]. This novel sensor also has been studied to detect other non-charged bitter substances for medicine, such as theophylline [55].

Monosodium L-glutamate (MSG) is commonly known as a flavoring enhancer of umami in food processing [56,57,58]. The pH of an MSG solution varies with different concentrations [59,60]. Iiyama et al. [61] found that the lipid/polymer membrane containing phosphate group exhibits a response to alkaline substances such as NaOH and NaHCO_3_ to the negative direction, closely resembling the response to MSG. It implies that the observed MSG response Is caused by the H^+^ dissociation from the phosphate group, i.e., the transfer of the phosphate group H^+^ of lipid molecule to the carboxy group of MSG. The commercialized umami sensor AAE, employing phosphoric acid di(2-ethylhexyl) ester (PAEE) as one of the lipids, similarly shows the negative-direction response to MSG. This response is not caused by the molecular recognition of chemical structure of MSG by the receptive membrane, but by the transfer of H^+^ of PAEE to the carboxy group of MSG. On the other hand, Kobayashi and other co-workers employed an AAE sensor for taste selectivity measurements and found that the sensor exhibits a notable response (greater than 10 mV) to astringent substances [62]. This finding raises concerns regarding the taste selectivity of umami sensor AAE, which is equipped with lipid PAEE, to differentiate between umami and astringent substances, especially at a low concentration of umami sample solution.

To address these challenges and enhance the taste sensor’s capability for detecting umami substances, a novel lipid is required to replace lipid PAEE. Additionally, the surface modification method used to measure non-charged bitter substances was considered useful for measuring umami substances. In this study, we conducted surface modification on lipid/polymer membranes for umami substances measurement, such as MSG and monosodium L-aspartate (MSA). Several modifies with obvious structural similarities were conducted, and concentration-dependence experiments were designed and carried out. By comparing and analyzing their experimental data, the structural conditions of the modifiers required to measure umami substances were summarized, and the modifier suitable for measuring umami substances was discussed. Furthermore, the taste sensors treated with the optimal modifier were validated for their selectivity to umami through a series of selectivity experiments spanning the five basic tastes and astringency.

## 2. Materials and Methods

### 2.1. Reagents

The lipid/polymer membrane comprises tetradodecylammonium bromide (TDAB) as the lipid, dioctyl phenyl-phosphonate (DOPP) as the plasticizer, and polyvinyl chloride (PVC) as the supporting material. Tetrahydrofuran (THF) was used as the organic solvent. TDAB and THF were purchased from Sigma-Aldrich (St. Louis, MO, USA). DOPP was acquired from Dojindo Molecular Technologies (Kumamoto, Japan), while PVC was obtained from FUJIFILM Wako Pure Chemical Corporation (Osaka, Japan).

Four types of modifiers were utilized, including terephthalic acid (TPA), 2-hydroxyterephthalic acid (2-HTA), 2,6-dihydroxyterephthalic acid (2,6-DHTA), and 2,6-DHBA. 2-HTA and TPA were procured from Tokyo Chemical Industry (Tokyo, Japan). 2,6-DHTA was sourced from BLDpharm (Shanghai, China), and 2,6-DHBA was acquired from FUJIFILM Wako Pure Chemical Corporation (Osaka, Japan).

**Figure 1:**
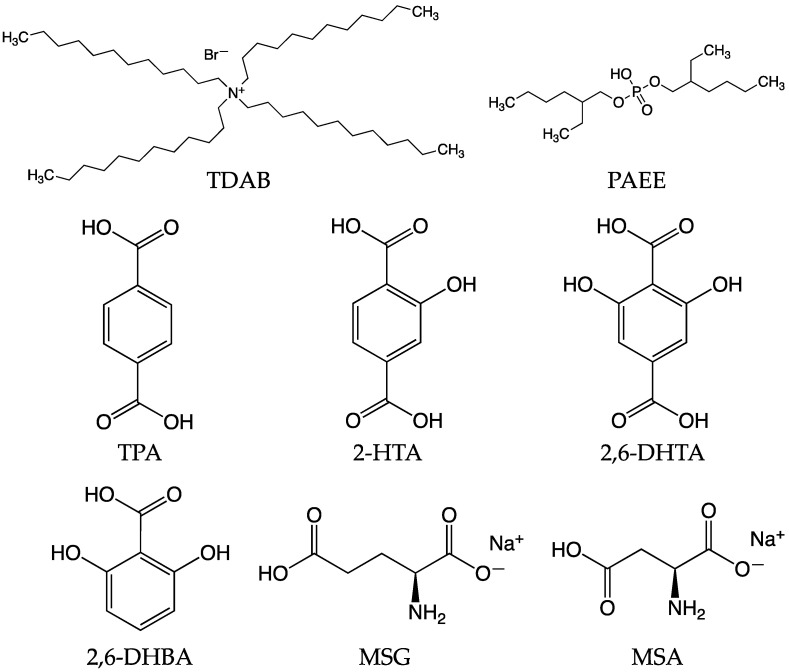
The structural formula of TDAB, PAEE, TPA, 2-HTA, 2,6-DHTA, 2,6-DHBA, MSG, and MSA.

MSG, potassium chloride (KCl), tartaric acid, quinine hydrochloride, tannic acid, and sucrose were purchased from Kanto Chemical Co. (Tokyo, Japan). MSA was purchased from FUJIFILM Wako Pure Chemical Corporation (Osaka, Japan). Figure 1 shows the structural formula of TDAB, PAEE, TPA, 2-HTA, 2,6-DHTA, 2,6-DHBA, MSG, and MSA.

### 2.2. Fabrication of Lipid/Polymer Membrane and Surface Modification

In this study, we prepared five types of sensor electrodes with lipid/polymer membranes including TDAB as a lipid. The process for fabricating lipid/polymer membranes is similar to a previous report [55]: Initially, a cleaned and dried 20 mL screw tube bottle was prepared for mixing lipid, DOPP, PVC, and THF. 0.01 mmol TDAB was dissolved in 10 mL THF. Subsequently, 1.5 mL DOPP and 800 mg PVC were added sequentially. The resulting mixture was stirred thoroughly and then spread onto a clean Petri dish (90 mm φ). The TDAB lipid/polymer membrane was formed through the evaporation of THF. The obtained lipid/polymer membrane was cut and placed onto a sensor electrode.

Three groups of sensor electrodes were immersed into 0.03 wt% of 2,6-DHBA, 2,6-DHTA, and 2-HTA aqueous solution each for surface modification for 72 h by the same method before [55]. We soaked a group of sensor electrodes into the reference solution containing 3.33 M KCl and 0.3 mM tartaric acid for 72 h as a reference group in the measurement.

Since TPA is insoluble in both water and organic solutions [63,64], it was directly incorporated into the membrane for preparing another group of sensor electrodes. To determine the optimal concentration of TPA in the membrane for MSG detection, we prepared four membranes, each containing different amounts of TPA, specifically 0.16, 1, 10, and 100 mg. TPA was initially placed into a cleaned 20 mL screw tube bottle, followed by the sequential addition of 10 mL THF, 0.01 mmol TDAB, 1.5 mL DOPP, and 800 mg PVC. The mix membrane solution was stirred well and gently spread onto the cleaned Petri dish (90 mm φ) to form the lipid/polymer membrane. After placing the membranes onto the sensor electrodes, they were immersed into a reference solution for 72 h.

### 2.3. Procedure of Taste Sensor Measurement

**Figure 2:**
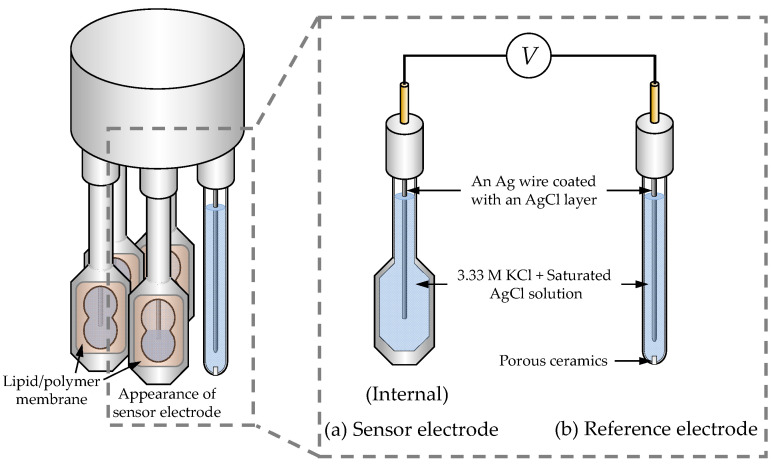
The structure of detection unit: (**a**) sensor electrode, and (**b**) reference electrode.

A TS-5000Z taste sensing system (Intelligent Sensor Technology, Atsugi, Japan) was utilized for all the taste sensor measurements. This system can accommodate two detection units, each including a reference electrode and up to four sensor electrodes. Figure 2 illustrates the structure of the detection unit. Both the sensor electrode and the reference electrode are equipped with an Ag wire coated with an AgCl layer and are filled with an inner solution containing KCl at a concentration of 3.33 M and saturated AgCl.

**Figure 3:**
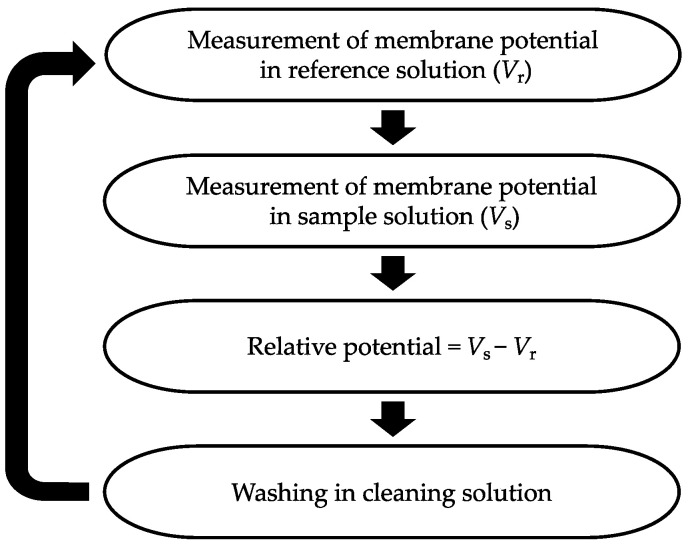
Procedure of taste sensor measurement.

Figure 3 illustrates the procedure for taste sensor measurements. Initially, the sensor electrodes and the reference electrode are immersed in a reference solution for 30 s to obtain the reference potential (*V*_r_). Subsequently, they are immersed in the sample solution for 30 s to obtain the sample potential (*V*_s_). The relative potential in the sample solution is calculated by taking the difference between *V*_s_ and *V*_r_. Finally, the sensor electrodes and the reference electrode are cleaned with a water-based solution consisting of 10 mM KOH, 100 mM KCl, and 30 vol% EtOH in preparation for the next measurement cycle. To ensure the reliability of the experimental data, we set the taste sensor to go through this detection step five times, and selected the data from the last three times out of the five for analysis.

### 2.4. Response Principle of the Taste Sensor

**Figure 4:**
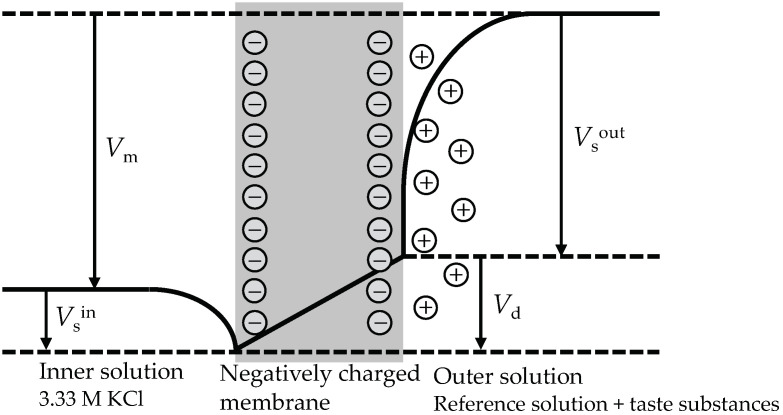
The potential profile of a negatively charged lipid/polymer membrane.

In a broad sense, the taste sensor are ion-selective electrodes fabricated with lipid/polymer membranes based on ion exchangers [46]. To elucidate the response mechanism of the taste sensor, the potential profile of a lipid/polymer membrane containing negatively charged lipid, plasticizer, and PVC is illustrated in Figure 4. The negatively charged lipid/polymer membrane acts as a barrier between two KCl solutions. When the concentrations of the solutions on either side of the membrane are different, the membrane potential difference appears between the outer and inner solutions.

The membrane potential comprises the surface potential generated at the aqueous interface of the membrane and the diffusion potential within the membrane [46]. Under constant temperature conditions, the inner solution potential remains constant. The outer solution, on the other hand, contains taste substances. The volume of the membrane and the amount of lipid molecules determine the molecular surface area occupied by each lipid molecule. This quantity, along with the concentration of the taste substance solution, determines the membrane surface charge density, subsequently influencing the outer solution potential.

For negatively charged lipid/polymer membranes, due to electrostatic interactions, cations are attracted near the membrane surface, while anions move away from the membrane surface. Consequently, a diffusion double layer is formed between negative charges on the membrane surface and cations in solution. The diffusion potential within the membrane is a potential difference caused by the dissimilarity in the mobility of cations and anions.

Therefore, the membrane potential *V*_m_ can be expressed as

*V*_m_ = *V*_s_^out^ + *V*_d_ − *V*_s_^in^, (1)

where *V*_s_^out^ and *V*_s_^in^ is the outer solution potential and the inner solution potential, respectively, and *V*_d_ is the diffusion potential.

These lipid/polymer membranes have ionic and/or hydrophobic (or hydrophilic) properties, as mentioned in the Introduction. The membrane can classify and quantify five basic taste qualities, which have ionic and/or hydrophobic (or hydrophilic) properties in a broad sense. There is scarcely a molecular recognition in this response mechanism, because the surface potential in itself is nonspecific in the strict sense.

In this study, we developed a novel type of lipid/polymer membrane by using modifiers which specifically interact with umami substances such as MSG and MSA. We chose four kinds of modifiers, i.e., 2,6-DHBA, 2,6-DHTA, TPA, and 2-HTA, for this purpose.

### 2.5. Measurement of Taste Samples by Fabricated Taste Sensors

All umami samples and taste quality samples were prepared using the reference solution (0.3 mM of tartaric acid and 30 mM of KCl aqueous solution) as the base. All five fabricated sensors, i.e., sensors modified with 2,6-DHBA, 2,6-DHTA, TPA, 2-HTA, and sensors without surface modification, were used for measuring umami samples. Sensors modified with 2-HTA and 2,6-DHTA were used for measuring various taste quality samples. Table 1 displays the composition of these taste quality samples.

## 3. Result and Discussion

### 3.1. Detection of Umami Substances Using Taste Sensors Treated with 2,6-DHBA and 2,6-DHTA

**Figure 5:**
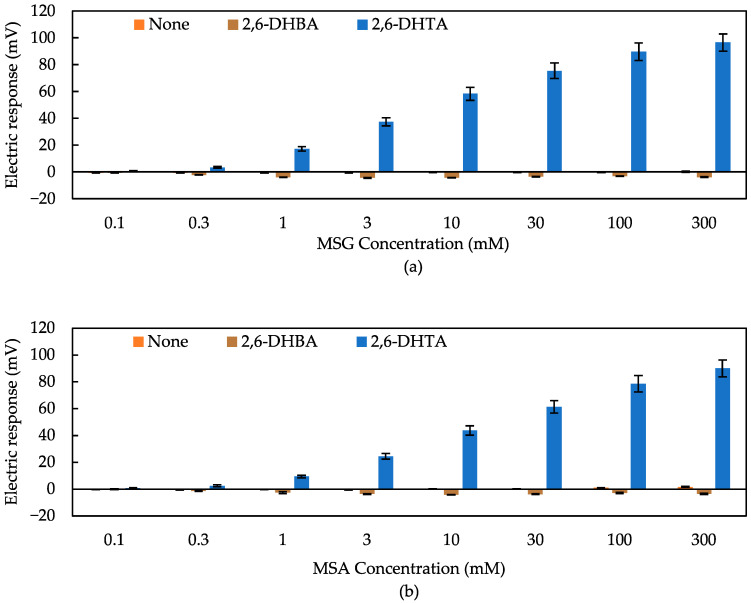
Response to (**a**) MSG and (**b**) MSA solutions with three types of sensors: the sensor without surface modification (correspond to data of ‘None’), and sensors modified with 2,6-DHBA and 2,6-DHTA, respectively. Error bars indicate the SD of data, *n* = 4 (electrode) × 3 (rotation) = 12 values.

First, we employed 2,6-DHBA and 2,6-DHTA as modifies to detect umami. These two materials were utilized to detect non-charged bitterness substances in previous studies [49,53,54,55]. In contrast to the structure of 2,6-DHBA, which features only one carboxyl group on the benzene ring, 2,6-DHTA contains two carboxyl groups on the para-position of the benzene ring. Figure 5 presents the response to MSG and MSA solutions with three sensors: the sensor without surface modification, the sensor modified with 2,6-DHBA, and the one with 2,6-DHTA. Mean values and standard deviations were calculated from 12 (4 electrodes × 3 rotations) sets of electrical response values. Comparing the data in Figure 5, it is evident that the taste sensor treated with 2,6-DHTA exhibited a significant response to MSG and MSA, while other sensors were unable to detect them. As the concentration of the umami sample solution increased, the response obtained by the 2,6-DHTA-treated sensor also increased. As shown in Figure 5 (orange bar), the sensor without surface modification showed negligible responses (less than 10 mV) to umami substances. These results indicate that the surface modification method using 0.03 wt% 2,6-DHTA as a modifier is a practical approach for the sensor to detect MSG. Furthermore, it is notable that the response values of the taste sensor treated with 2,6-DHTA to these umami substances were consistently positive, and the values increased according to the MSG concentration. MSG ionized in aqueous solution is electrically negative; hence, the positive response of this sensor cannot be considered to be a simple interaction between the membrane and ionized MSG.

The 2,6-DHBA-treated sensor has proven effective in detecting non-charged bitter substances [49,53,54,55], while it was insensitive to MSG and MSA, as shown in Figure 5. Given the ineffectiveness of the 2,6-DHBA-treated sensor in detecting MSG and MSA, and the remarkable sensitivity of the 2,6-DHTA-treated sensor, it suggests that the structure of 2,6-DHTA, which features two carboxyl groups on the para-position of the benzene ring, is suitable for detecting umami substances.

### 3.2. Effect of Intramolecular H-Bonds within Modifier for Umami Substances Detection

To investigate the effect of intramolecular H-bonds within modifiers on the sensitivity of the taste sensor to MSG, we utilized two other modifiers, TPA and 2-HTA, in addition to 2,6-DHTA. As shown in Figure 1, 2-HTA contains one hydroxyl group, 2,6-DHTA contains two hydroxyl groups, while TPA does not contain any hydroxyl group. The hydroxyl group on the benzine ring are capable of forming an intramolecular H-bond with the adjacent carboxyl group [65,66,67].

**Figure 6:**
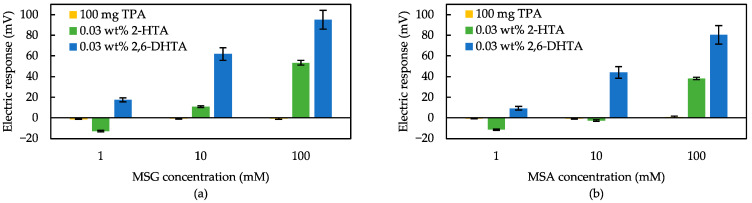
Response to (**a**) MSG and (**b**) MSA in sample solutions measured using three sensors with different modifiers. Yellow: the sensor membrane was fabricated mixed with 100 mg of TPA. Green: the sensor was soaked in 0.03 wt% 2-HTA solution for surface modification. Blue: the sensor was soaked in 0.03 wt% 2,6-DHTA solution. The error bar indicates the SD of data, *n* = 4 (electrode) × 3 (rotation) = 12 values.

Figure 6 shows the response to MSG and MSA solutions measured using sensors modified with TPA, 2-HTA, and 2,6-DHTA each. According to Figure 6, the 100 mg TPA-contained sensor showed negligible responses (<10 mV) to MSG and MSA. The similar result was obtained for sensors containing other different quantities of TPA, indicating that the TPA-contained sensor is unable to detect umami substances. The response of the 2,6-DHTA-treated sensor to 100 mM MSG (i.e., 95 mV) was nearly double the response obtained by the 2-HTA-treated sensor (i.e., 53 mV). The tendency of higher sensitivity in the 2,6-DHTA-treated sensor compared to the 2-HTA-treated sensor remained consistent, even when the sample was MSA or at the low concentrations. These results indicate that the number of intramolecular H-bonds between the carboxyl group and hydroxyl group in the molecular structure is crucial for enhancing the sensor sensitivity. This result is consistent with the earlier findings for caffeine sensors, indicating that the number of intramolecular H-bonds in the modifier molecules influences sensitivity to the sample [49].

### 3.3. Measurement of Various Taste Samples Using Sensors Treated with 2,6-DHTA and 2-HTA

**Figure 7:**
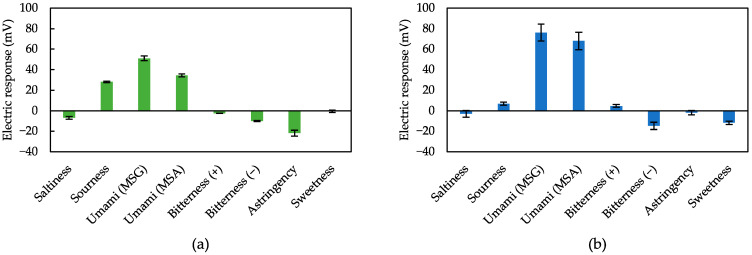
Response to the five basic tastes and astringency samples measured using sensors modified with (**a**) 2-HTA and (**b**) 2,6-DHTA, respectively. The error bar indicates the SD of data, *n* = 8 (electrode) × 3 (rotation) = 24 values.

We measured various taste samples to validate the effectiveness of 2,6-DHTA and 2-HTA-modified sensors as umami sensors. Selectivity, i.e., the ability of the sensor to respond strongly to umami and not to other tastes is important for practical applications. Figure 7a,b show the response to five basic tastes and astringency sample solutions using sensors modified with 2-HTA and 2,6-DHTA, respectively. Both the sensors treated with 2-HTA and 2,6-DHTA exhibited selectivity to MSG and MSA.

Taste sensors treated with 2-HTA exhibited a 20 mV response to the sourness sample. The dissociation of carboxyl groups not adjacent to the hydroxyl group of 2-HTA may have decreased in the acid pH domain, leading to an increase in membrane potential. Actually, the fact that the sensors respond to umami and sourness is not very important, because MSG is the sodium salt of glutamic acid, and then umami is not felt in the acid pH domain but felt around neutral pH [68].

Moreover, the taste sensor treated with 2,6-DHTA showed significant responses to MSG and MSA, while it demonstrated negligible response (less than 10 mV) to saltiness, sourness, bitterness (+), and astringency. Response values for bitterness (−) and sweetness are not necessarily negligible, but they have no impact on identifying umami substances since they exhibited opposite responses to umami substances.

2,6-DHTA is practical surface modification substance for taste sensors in umami substances measurement, showing good selectivity for MSG and MSA. Although 2,6-DHTA has been studied for caffeine detection [55], it is worth noting that 2,6-DHBA exhibits minimal response to MSG, whereas 2,6-DHTA shows a significant response. Therefore, the simultaneous use of these two modifiers can effectively differentiate between umami substances and caffeine.

### 3.4. Mechanism Speculation of Positive Response Value of Taste Sensor Treated with 2,6-DHTA to MSG

As found in Figure 7, the developed sensor modified with 2,6-DHTA responds specifically to umami substances, MSG and MSA. The conditions for modifiers required to measure MSG and MSA are summarized as follows: (1) Two carboxyl groups on the para-site of the benzene ring are essential; (2) the presence of hydroxyl groups that form intramolecular H-bonds with the adjacent carboxyl groups are also essential. Moreover, an increase in the number of intramolecular H-bonds corresponds to an increase in the sensor’s sensitivity to the substances.

Additionally, the distance between the carbon atoms of the two carboxyl groups in 2,6-DHTA is 6.160 Å, while in MSG it is 5.335 Å (calculated from Marvin 23.7.0, Che-mAxon, Budapest, Hungary). These observations suggest a possibility of intermolecular interactions between 2,6-DHTA and MSG.

**Figure 8:**
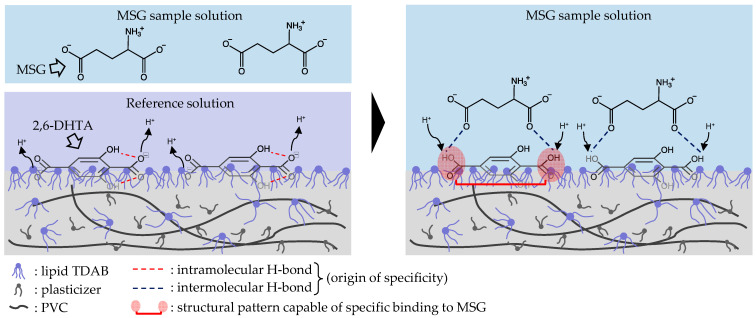
Estimated binding structure between 2,6-DHTA and MSG.

Therefore, we propose a detection mechanism to explain the response to MSG (Figure 8). 2,6-DHTA formed intramolecular H-bonds between its carboxyl groups and hydroxyl groups by dissociating H^+^ into the reference solution. When the 2,6-DHTA-treated sensors were immersed in the MSG sample solution, intermolecular H-bonds were formed in the two places between the carboxyl groups of 2,6-DHTA and the carboxyl groups of MSG, altering the dissociation of 2,6-DHTA on the lipid/polymer membrane. The two intramolecular H-bonds of 2,6-DHTA were disrupted. The formation and disruption of H-bonds resulted in positive change in the membrane potential associated with taking back H^+^ to the carboxyl groups of 2,6-DHTA. This process is described as the transition ‘intra- to intermolecular H-bonds’.

As shown in Figure 1, MSA has one less carbon atom than MSG, resulting in its length shorter than MSG. Consequently, we speculate that the shorter length of MSA leads to relatively less stability in its binding with 2,6-DHTA. As illustrated in Figure 7, the response of MSA is slightly lower than that of MSG. This suggests that MSG’s size is relatively well-matched with 2,6-DHTA. Further research is required to investigate the taste sensor treated with 2,6-DHTA to distinguish between MSG and amino acid substances of a similar size to MSG, e.g., glutamine, which has no umami taste. Moreover, we will employ chemical analysis techniques, e.g., nuclear magnetic resonance (NMR) methods to verify this mechanism conclusively.

## 4. Conclusions

In conclusion, this study demonstrated that the taste sensor modified with terephthalic acid analogs detects umami substances, including MSG and MSA. The taste sensor treated with 2,6-DHTA had high sensitivity for the umami substances MSG and MSA, while the response to other taste substances was negligible; hence, the taste sensor treated with 2,6-DHTA is expected to be applied to practical umami sensors. It is also important to note that the sensor response was positive even though umami substances ionized negatively in aqueous solution. Furthermore, through investigation using some terephthalic acid analogs as modifiers, we identified both the two carboxyl groups on the para-position of the benzene ring and the hydroxyl group next to the carboxyl group for intramolecular H-bond formation are the essential structural characteristics for detecting MSG and MSA. The number of intramolecular H-bonds positively correlates with changes in membrane potential, emphasizing the importance of this feature. More research is needed to confirm the mechanism behind the positive response of 2,6-DHTA-treated taste sensors to MSG, such as employing NMR measurements to analyze the molecular interactions between 2,6-DHTA and MSG. Future work should explore sensitivity for other umami substances, such as IMP and GMP, to broaden the scope of applications for taste sensors with surface-modified membranes. This study showcases the potential of taste sensors and surface modification techniques in the realm of taste substance measurement and encourages further investigations in this field.

## Figures and Tables

**Table 1:** Composition of five basic tastes and astringency sample.

Sample	Composition	Concentration
Sourness	Tartaric acid	3 mM
Bitterness (+)	Quinine hydrochloride	0.1 mM
Bitterness (−)	Iso-α acid	0.01 vol%
Saltiness	Potassium chloride (KCl)	300 mM
Astringency	Tannic acid	0.05 wt%
Sweetness	Sucrose	1 M
Umami	Monosodium L-glutamate (MSG)	100 mM
	Monosodium L-aspartate (MSA)	

## Data Availability

Data are available on request.
